# The gut microbiome–bile acid-FXR interplay: a pivotal axis in metabolic and gastrointestinal diseases

**DOI:** 10.1080/19490976.2026.2665890

**Published:** 2026-05-01

**Authors:** Jiawen Shou, Ting Fu

**Affiliations:** a Pharmaceutical Sciences Division, School of Pharmacy, University of Wisconsin–Madison, Madison, WI, USA; b University of Wisconsin Carbone Cancer Center (UWCCC), Madison, WI, USA

**Keywords:** Bile acid, gut microbiota, metabolism, farnesoid X receptor

## Abstract

The gut microbiota is increasingly recognized as an essential metabolic “organ”, involved not only in nutrient extraction and energy metabolism but also in generating diverse bioactive metabolites. Among these metabolites, bile acids (BAs) - initially synthesized in the liver - are substantially modified by bacterial enzymes in the gut, enabling them to engage various host signaling pathways. Notably, these BAs interact with critical host receptors, such as nuclear farnesoid X receptor (FXR) and G protein-coupled BA receptor 1 (TGR5), influencing numerous metabolic processes. Given the complexity and significance of BAs signaling between microbiota-host interactions, a comprehensive review of this interplay is essential. Here, we systematically explore the molecular mechanisms underlying the BA-microbiota axis, emphasizing its role in metabolic, gastrointestinal, and immune-related diseases, with a focus on the roles of FXR signaling pathways.

## 1. Introduction

The gut microbiota, often regarded as a metabolic and signaling “organ,” profoundly influences host metabolism, physiology, and immunity through its dynamic interactions with host-derived and diet-derived molecules. Central to this dialogue are microbiota-produced or microbiota-modified metabolites, such as lipopolysaccharides, short-chain fatty acids (SCFAs), branched-chain amino acids (BCAAs), hormones such as oestrogens, and BAs.^1^ Among these, BAs have emerged as critical molecular messengers owing to their structural diversity and dynamics, bioactive properties, and extensive reciprocal interplay with the gut microbiota.

BAs, first structurally elucidated in 1932, have been characterized as amphipathic, detergent-like molecules synthesized from cholesterol within hepatocytes.^2^ Following their hepatic synthesis via classical and alternative biosynthetic pathways, BAs undergo conjugation with glycine (in humans) or taurine (in rodents), which are stored in the gallbladder. Postprandial release of BAs into the intestinal lumen facilitates lipid emulsification and nutrient absorption, with approximately 95% subsequently reabsorbed via enterohepatic circulation.^3^ Given their potent bioactivity, the synthesis, secretion, and recirculation of BAs are tightly controlled by dedicated receptors – most notably the farnesoid X receptor (FXR), which serves as the master regulator of BA homeostasis.^4^

Beyond their canonical role in lipid digestion, BAs are now well-recognized as endocrine signaling molecules that modulate a broad spectrum of metabolic and physiological processes. The synthesis, pool size, and composition of BAs are highly responsive to dietary inputs, particularly high-fat diets (HFDs), which can elevate circulating BA concentrations and thereby contribute to the onset and progression of metabolic disorders.^4^ Through interaction of the FXR, the G protein-coupled membrane BA receptor 1 (TGR5), and other BA-sensing receptors, BAs regulate glucose metabolism, insulin sensitivity, and inflammatory signaling pathways.^4^ Consequently, disturbances in BA signaling have been mechanistically linked to the pathogenesis of insulin resistance, obesity, diabetes, metabolic-associated fatty liver disease (MAFLD), metabolic-associated steatohepatitis (MASH), and hepatocellular carcinoma (HCC).

Contemporary research has further highlighted the bidirectional relationship between BAs and the gut microbiota. While the small intestine contains relatively sparse microbial populations (fewer than 10⁵ bacteria per gram of content), the colon harbors a dense and diverse community, often exceeding 10¹¹ bacterial cells per gram.^5^ Gut microbes profoundly reshape the composition of the BA pool through enzymatic biotransformation, generating a spectrum of microbiome-modified secondary and tertiary BAs with distinct biological activities. Several of these microbial BAs can enter the systemic circulation and exert extraintestinal effects, thereby influencing whole-body metabolic and immune homeostasis. Conversely, BAs themselves impose selective pressures on the composition and function of the gut microbiota, establishing a dynamic and reciprocal regulatory axis. Perturbations in this BA-microbiota interplay, arising from dietary patterns, antibiotic use, or other environmental influences, are increasingly recognized as contributing factors in a range of pathologies, including inflammatory bowel disease (IBD), colorectal cancer (CRC), and *Clostridioides difficile* infection (CDI).

Given the complex yet pivotal role of BAs as signaling molecules at the host–microbiota interface, this review systematically examines the biology of BAs and their dynamic interactions with gut microbes. We first provide an overview of primary BA synthesis and regulation, followed by an in-depth discussion of how the gut microbiota modifies and diversifies the BAs pool. We then explored the local and systemic effects of microbiota-modified BAs, with an emphasis on key signaling pathways involving the FXR and TGR5 receptors. Building on these mechanistic insights, we highlight how the microbiota-BA axis contributes to the pathogenesis of metabolic disorders, liver diseases, immune dysregulation and cancer, focusing on shared molecular mechanisms across these conditions. Finally, we discuss emerging therapeutic strategies targeting BA signaling and the gut microbiota, including pharmacological modulators, dietary interventions, and microbiota-based therapies, and outline future directions and challenges in this rapidly evolving field.

## 2. Bile-ology

### 2.1. Primary BA synthesis and regulation of the host BA pool

BAs are synthesized in hepatocytes through cytochrome P450 (CYP)-mediated oxidation of cholesterol, proceeding via two biosynthetic routes: the classical (or neutral) pathway and the alternative (or acidic) pathway.^3^ The classical pathway, responsible for at least 75% of BA production under normal physiological conditions, is primarily mediated by two cholesterol hydroxylase enzymes: CYP7A1 and CYP8B1, with CYP7A1 serving as the rate-limiting enzyme.^6^ The alternative pathway is initiated by CYP27A1 and further processed by CYP7B1.^6^

The predominant primary BAs synthesized in human hepatocytes are cholic acid (CA) and chenodeoxycholic acid (CDCA).^7^ In humans, ursodeoxycholic acid (UDCA) is classified as a secondary BA, produced by gut microbial transformation of CDCA in the colon.^8^ By contrast, in rodents, the spectrum of primary BAs is broader and includes CA, *α*-muricholic acid (αMCA), *β*-muricholic acid (βMCA), and UDCA, all of which are synthesized directly in the liver – making UDCA a primary BA in this context.^3^ Under physiological conditions, free (unconjugated) BAs are rarely found in bile. Instead, in the liver, primary BAs are conjugated with either taurine (predominantly in mice) or glycine (primarily in humans) by the enzymes bile acid-CoA synthase (BACS) and bile acid-CoA: amino acid *N*-acyltransferase (BAAT).^3^ Owing to their detergent-like properties, BAs can disrupt cellular membranes and damage organelles if not properly regulated.^9^ Conjugation not only increases the solubility of these hydrophobic molecules but also significantly reduces their cytotoxic potential.^10^ Conjugated BAs are actively secreted into bile via the bile salt export pump (BSEP) and subsequently stored in the gallbladder. In the postprandial state, the gallbladder contracts in response to hormonal signals such as cholecystokinin, releasing bile into the duodenum. The bile consists of mixed micelles formed by BAs, cholesterol, and phospholipids, which are essential for the emulsification of dietary fats. These micelles lower surface tension and break down large fat droplets into smaller emulsified particles, enhancing lipid digestion and absorption.

**Figure 1.**
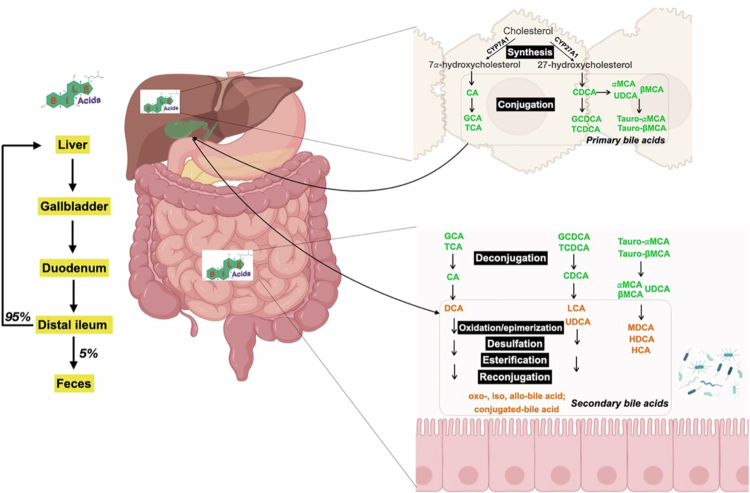
BA synthesis and enterohepatic circulation. Primary BAs, CA, and CDCA are synthesized in the liver from cholesterol. In rodents, CDCA is further converted into αMCA, βMCA, and UDCA. Before secretion, BAs are conjugated with either taurine (predominantly in mice) or glycine (primarily in humans), then incorporated into bile, stored in the gallbladder, and released into the intestinal lumen. Upon exposure to the gut microbiota, primary BAs are deconjugated to release taurine or glycine. Both conjugated and deconjugated BAs can then undergo microbial transformations, including oxidation/epimerization, desulfation, esterification, and reconjugation, leading to the formation of secondary BAs. More than 95% of BAs are reabsorbed in the ileum and recycled back to the liver via enterohepatic circulation.

Upon exposure to the gut microbiota, a portion of primary bile acids undergoes deconjugation, releasing taurine or glycine. After lipid absorption, both conjugated and unconjugated BAs are predominantly reabsorbed in the distal ileum via the apical sodium-dependent bile acid transporter (ASBT), which is localized on the microvillus membrane of intestinal epithelial cells (IECs).^11^ Within the enterocyte, BAs bind to the ileal bile acid-binding protein (IBABP), which facilitates their intracellular transport toward the basolateral membrane.^12^ The heteromeric organic solute transporter complex, which is composed of organic solute transporter α/β (OSTα and OSTβ), mediates the export of BAs into the portal circulation.^13^ Subsequently, BAs return to the liver, where they are primarily taken up by hepatocytes through the sodium taurocholate cotransporting polypeptide (NTCP) and secondarily by organic anion-transporting polypeptides (OATPs).^14^ Once reabsorbed, BAs may undergo re-conjugated if necessary and re-secreted into bile, thus completing the enterohepatic circulation.^11^ This highly efficient recycling process enables the reabsorption of approximately 95% of BAs, thereby playing a critical role in maintaining BAs homeostasis. Figure 1 summarized the synthesis and enterohepatic circulation of BAs.

### 2.2. Microbial modification and diversification of BAs

The gut harbors a diverse and dynamic community of symbiotic bacteria and other microorganisms, collectively known as the gut microbiota. As BAs transit through the gastrointestinal tract, they engage in an inevitable and bidirectional interplay with these microbes. In concert with the host, the gut microbiota co-produces a wide array of metabolites that function as key signaling molecules and energy substrates, thereby profoundly shaping host physiology. Extensive studies have demonstrated that the gut microbiota metabolizes primary BAs into secondary and tertiary BAs through a range of biochemical transformations, including deconjugation, oxidation and epimerization, desulfation, esterification, and reconjugation, thus expanding the structural diversity and hydrophobicity of the BA pool^15^ (Figure 1). Conversely, BAs actively modulate the composition, diversity, and metabolic activity of the gut microbiome.^15^ Together, these reciprocal interactions play a central role in regulating the overall BA pool, maintaining metabolic and immune homeostasis, and preserving normal intestinal epithelial turnover and barrier function.

**Deconjugation:** Deconjugation of glycine- (glyco-) or taurine- (tauro-) conjugated BAs, catalyzed by the enzyme bile salt hydrolase (BSH),^3^ represents the initial and essential step for all subsequent microbial modifications. Importantly, deconjugation prevents active reabsorption of BAs in the small intestine via ASBT,^16^ thereby increasing the pool of BAs available for further microbial modification in the colon. BSH, encoded by the highly conserved bsh gene across major gut phyla, including *Bacteroidetes*, *Firmicutes*, and *Actinobacteria*,^17^^,^^18^ cleaves the amide bond to release the amino acid moiety. BSH activity is thought to protect bacteria from the toxicity of conjugated BAs, while the released amino acids may serve as additional nutrient sources, supporting bacterial survival and fitness.

**Dehydroxylation:** Deconjugated BAs can undergo further biotransformation by the gut microbiota. A key modification involves the removal of the hydroxyl group at the C-7 position of the steroid nucleus, a reaction catalyzed by bacterial 7α- or 7β-dehydroxylases. These enzymes are encoded by the polycistronic bile acid-inducible (bai) operon, which is primarily found in *Clostridium* species (notably clusters XIVa and XI) and *Eubacterium*, both members of the phylum *Firmicutes*.^19^^,^^20^ The bai operon comprises multiple genes (e.g., baiB, baiCD, baiE, baiF, baiG, and baiH), which are co-regulated and act in concert to mediate multistep dehydroxylation reactions.^21^ Through this transformation, CA and CDCA are converted into the secondary BAs-deoxycholic acid (DCA) and lithocholic acid (LCA), respectively, thereby significantly increasing the hydrophobicity and altering the overall composition of the BAs pool.^22^

**Oxidation and epimerization:** The structural diversity of the BAs pool is further expanded by the gut microbiota through oxidation and subsequent epimerization of hydroxyl groups at the C-3, C-7, and C-12 positions on the steroid nucleus.^23^ These transformations are primarily mediated by bacterial hydroxysteroid dehydrogenases (HSDHs), a class of enzymes broadly distributed among several bacterial phyla, including *Actinobacteria*, *Proteobacteria*, *Firmicutes*, and *Bacteroidetes*.^24-26^ HSDHs catalyze reversible interconversions between hydroxyl and keto groups, with the direction of conversion determined by the local redox environment. For instance, 7α-HSDH catalyzes the oxidation of CDCA to 7-keto-lithocholic acid (7-keto-LCA), which can subsequently be reduced by 7β-HSDH to form UDCA.^27^ Additionally, recent studies have demonstrated that certain human gut bacteria expressing 5α-reductases are capable of modifying classical 5β-bile acids by reducing the C-5 position of the steroid core, thus generating allo- (or 5α-) bile acid epimers and further contributing to BAs structural diversity.^28^

**Esterification and desulfation:** Esterification of BAs – observed in genera such as *Bacteroides*, *Eubacterium*, and *Lactobacillus* – can increase the hydrophobicity of BAs and decrease their solubility.^22^ For instance, the esterification of CA with succinic acid has been identified in *Bacteroides uniformis.*^29^ In addition, bile acid sulfatase activity has been detected in bacterial genera such as *Clostridium*, *Peptococcus*, *Fusobacterium*, and *Pseudomonas*, where it plays a key role in the desulfation of sulfated BAs.^30^^,^^31^ This microbial process regenerates unconjugated BAs, increasing their hydrophobicity and potential signaling activity. While host-mediated sulfation enhances BAs solubility and facilitates BA excretion as a detoxification mechanism, microbial desulfation may counteract this effect and contribute to the reactivation of BAs signaling in the gut.^32^

**Non-canonical amino acid conjugation:** Primary BAs are typically conjugated with glycine or taurine at the C24 acyl site prior to hepatic secretion, a modification that enhances their solubility and supports efficient enterohepatic circulation. Interestingly, recent discoveries have introduced a significant paradigm shift: BAs can also undergo amide conjugation with aromatic and branched-chain amino acids such as methylcysteamine,^33^ phenylalanine,^34^ tyrosine,^35^ and leucine.^34^ Remarkably, these non-canonical conjugations are mediated not by the host but by the gut microbiota, uncovering a previously unrecognized layer of microbial influence on BAs chemistry and signaling.

**Secondary ecological effects driven by BA:** Beyond serving as substrates for microbial enzymes, BAs function as ecological selectors, favoring taxa that tolerate or transform specific BA chemistries and thereby restructuring community metabolic networks. Shifts toward hydrophobic or conjugated BA pools (e.g. elevated DCA, LCA, or microbially conjugated BAs) narrow ecological niches, enrich bai-operon–bearing *Clostridium*/*Eubacterium spp.*, and modulate signaling microenvironments that feed back on host FXR/TGR5 pathways.^33^^,^^36-39^ These secondary community alterations further influence epithelial barrier function, immune polarization, and systemic metabolism – amplifying BA effects beyond first-order enzymatic transformations.

### 2.3. BA receptors and signaling mechanisms

#### 2.3.1. BAs and their receptors in various cell types

BAs function not only as digestive agents but also as signaling molecules that regulate key physiological processes in the liver, intestine, and immune system by binding to specific receptors. These receptors are broadly classified into two categories: nuclear receptors and membrane-bound receptors. The key nuclear receptors that interact with BAs include FXR (NR1H4), pregnane X receptor (PXR; NR1I2), vitamin D receptor (VDR; NR1I1), and constitutive androstane receptor (CAR; NR1I3).^9^ Membrane-bound G protein-coupled receptors include TGR5 (GPBAR1) and sphingosine-1-phosphate receptor 2 (S1PR2).^9^

Among these, FXR and TGR5 are the most extensively studied and serve as central regulators in BAs signaling pathways. **FXR** is highly expressed in the liver, intestine, kidneys, adrenal glands and innate immune cells (such as macrophage).^40^ The binding affinity of BAs for FXR varies across species, with CDCA showing the strongest activation, followed by weaker agonists such as CA and LCA.^41^ In mice, certain BAs—including tauro-*α*-muricholic acid (T-αMCA), tauro-*β*-muricholic acid (T-βMCA), DCA, and to a lesser extent UDCA – can act as FXR antagonists, while DCA and UDCA may also display weak agonistic properties in humans.^42^^,^^43^ Conjugated BAs generally exhibit weaker FXR activation.^44^ Activation or inhibition of FXR regulates BAs hemostasis, lipid and glucose metabolism, epithelial cell proliferation, and inflammatory responses.^9^^,^^15^ **TGR5** is primarily expressed in the liver (notably in Kupffer cells), intestine (particularly in enteroendocrine cells), brown and white adipose tissues (with higher expression in brown adipose tissue), and various immune cells (with higher expression in innate immune cells).^45^ TGR5 exhibits a distinct BA activation profile compared to FXR. The most potent natural activator of TGR5 is taurolithocholic acid (TLCA), followed by LCA, conjugated DCA, and DCA. Other BAs, such as CDCA, UDCA, and CA are much weaker TGR5 agonists.^46^^,^^47^ TGR5 exhibits largely conserved ligand specificity and functional roles in both humans and mice.^46^^,^^47^ Activation of TGR5 can lead to improved insulin sensitivity, enhanced energy expenditure, and anti-inflammatory effects.^9^

**PXR**, mainly expressed in the liver and intestine, functions as a key regulator of xenobiotic and endobiotic detoxification pathways.^48^ LCA and its derivative, 3-oxo-LCA, have been shown to activate PXR, thereby contributing to protection against LCA-induced cholestatic liver injury disease.^49^ **VDR** is highly expressed in the liver, intestine, kidney, and bone, as well as a variety of immune cells. Classically, VDR is activated by 1,25-dihydroxyvitamin D₃, the active form of vitamin D, and regulates calcium and phosphate homeostasis, bone metabolism, and immune function. Beyond its classical role, the VDR also functions as a BA sensor, responsive to certain secondary BAs such as LCA and 3-oxo-LCA, suggesting potential protective effects against immune diseases and cancer.^41^ Notably, VDR signaling plays a critical role in shaping the RAR-related orphan receptor gamma (RORγ)-dependent transcriptional program in colonic regulatory T cells (Tregs).^50^ **CAR** is widely expressed in the liver, intestine, and other peripheral tissues, such as the brain.^51^ Functionally, the CAR acts as a key sensor and regulator of both endogenous metabolites and xenobiotic compounds.^52^ Among BAs, the LCA and its derivatives have been identified as potent CAR activators.^53^ Like PXR, the activation of CAR modulates the expression of genes involved in BA detoxification and transport, thereby contributing to the maintenance of BAs homeostasis. Importantly, recent studies have shown that CAR directs T cell adaptation to BAs in the small intestine.^54^ **S1PR2** broadly expressed in the liver, immune cells, vasculature, and several other organs and mediates a variety of cellular responses, including cell proliferation, migration, inflammation, and maintenance of vascular integrity.^55^ Conjugated BAs, such as TCA, can activate S1PR2 to regulate hepatic lipid metabolism.^56^

Beyond the classical BA receptors, emerging studies have demonstrated that BAs can also modulate additional targets, including the RORγ,^50^ nuclear hormone receptor (NR4A1),^28^ androgen receptor (AR),^57^ muscarinic receptor,^58^ and formyl peptide receptors (FPR).^59^ **RORγt,** a key isoform of the RAR-related orphan receptor gamma, is highly expressed in immune cells, particularly in T helper 17 (Th17) cells and type 3 innate lymphoid cells (ILC3s), where it acts as the master regulator of their development and the production of IL-17 family cytokines.^60^ Oxysterols, cholesterol biosynthesis intermediates, are widely considered the natural ligands for RORγt.^61^ Recent studies have demonstrated that certain microbial BAs, notably 3-oxo-LCA, can directly bind to and inhibit RORγt activity, thereby modulating Th17 cell differentiation and mucosal immune responses.^62^ **NR4A1**, an orphan nuclear hormone receptor broadly expressed in metabolic and immune tissues, has recently been identified as a BA-responsive receptor. The activation of NR4A1 by isoalloLCA is critical for regulating Treg cell function and modulating immune responses during intestinal inflammation.^28^ **AR** is primarily expressed in reproductive tissues and regulates genes involved in male sexual development and muscle growth, by binding androgens such as testosterone and dihydrotestosterone.^63^ A recent study noted that gut microbiota-derived BAs, including derivatives of 3-oxo-LCA, act as potent AR antagonists.^57^ The **muscarinic M3 receptor** is a key component of the parasympathetic nervous system, regulating physiological functions in smooth muscle (including the gastrointestinal tract and bronchi), glands, and the vascular endothelium. Notably, glycine- or taurine-conjugated DCA has been identified as a natural antagonist of the M3 receptor.^64^ **FPRs** are predominantly expressed on immune cells such as neutrophils and monocytes, where they play a central role in host defense and inflammation by mediating the chemotaxis of immune cells to sites of infection or tissue injury.^65^ Notably, CDCA has been identified as an endogenous antagonist of FPR.^66^

Despite significant progress in elucidating BAs, their receptors, and downstream signaling pathways, important questions remain regarding receptor specificity, ligand selectivity, and the determinants of tissue-specific responses. The growing discovery of novel microbial BAs highlights the complexity of BAs as microbiota-derived messengers, capable of signaling to diverse and distant organs. As such, it is highly likely that additional BA receptors will be identified, and that established “master” receptors may be found to have novel functions in distinct cell types and tissues. Continued research in this area promises to expand our understanding of the multifaceted roles of BAs in host physiology and interorgan communication.

**BA signaling mechanisms:** FXR is a master regulator of BAs homeostasis in both the liver and gut, orchestrating BAs synthesis, transport, secretion, and absorption. In hepatocytes, BAs-activated FXR induces the expression of small heterodimer partner (SHP), which interacts with liver receptor homolog-1 (LRH-1) to suppress the transcription of CYP7A1, the rate-limiting enzyme in the classical BAs synthesis pathway.^67^ FXR activation also enhances BAs excretion by upregulating efflux transporters, including the BSEP and multidrug resistance-associated protein 2 (MRP2),^68^ while simultaneously inhibiting hepatic BAs reuptake by downregulating basolateral uptake transporters such as NTCP, OATP1B1, and OATP1B3.^45^ In the distal ileum, FXR is activated by agonistic BAs and induces the expression of fibroblast growth factor 15 (Fgf15) in mice (or FGF19 in humans). FGF15/19 enters the portal circulation and binds to fibroblast growth factor receptor 4 (FGFR4) in complex with *β*-Klotho in the liver, triggering the extracellular signal-regulated kinases (ERK) 1/2 cascade, which further represses CYP7A1 expression.^68^ Intestinal FXR activation also facilitates BAs elimination by upregulating IBABP, which promotes apical-to-basolateral BAs transport within enterocytes and by inducing OSTα/OSTβ to accelerate BAs efflux into the portal circulation.^69^ Additionally, FXR suppresses the expression of the apical sodium-dependent bile acid transporter (ASBT) at the ileal brush border cells via a SHP-dependent manner,^70^ thereby limiting BAs uptake into enterocytes and controlling the physiological levels of BAs within enterocytes.

Beyond its central role in maintaining BAs homeostasis, FXR also regulates lipid and glucose metabolism, hepatic regeneration, autophagy, and intestinal microbial composition. While these functions have been extensively reviewed elsewhere, the present review focuses specifically on how microbial BAs regulate FXR signaling. For example, depletion of the gut microbiota, either in germ-free mice or in animals treated with broad-spectrum antibiotics,leads to significant dysregulation of BAs metabolism compared to conventionally raised animals. This dysregulation disrupts FXR-dependent pathways – including the CYP7A1, FGF15, BSEP, MRP2, MDR3, and ABCG5/G8 – thereby altering BAs synthesis, transport, and enterohepatic circulation, and ultimately impairing feedback regulation of the BAs pool.^71^^,^^72^ Moreover, in certain disease models, such as IBD and CRC, changes in the gut microbiota composition reduce the capacity for BAs deconjugation. This can lead to the accumulation of a primary BA, tauro-*β*-muricholic acid (T-βMCA), a potent FXR antagonist in mice, which in turn, diminishes FXR signaling and further perturbs BAs homeostasis.^73^^,^^74^

In addition to FXR, other host receptors' signaling is directly influenced by microbial BAs. For example, gut microbiota depletion reduces the availability of microbial BAs such as DCA and LCA that activate TGR5, resulting in diminished TGR5 signaling and increased susceptibility to IBD and metabolic dysfunction.^75^^,^^76^ Similarly, decreasing the abundance of BSH-producing bacteria can alter PXR and CAR activity, thereby modulating BAs homeostasis.^77^

## 3. Role of the gut microbiota-BA axis in disease pathogenesis

The gut microbiota-BAs axis has emerged as a dynamic regulator of host health, exerting profound effects on metabolic, gastrointestinal, and immune-related diseases. Recent advances have revealed that gut microbiota-derived BAs act as powerful modulators of host physiology, not only through classical digestive functions but also by finely tuning key signaling pathways. This review specifically highlights how microbial BAs regulate downstream receptors’ signaling, offering exciting new perspectives on disease pathogenesis and therapeutic opportunities.

### 3.1. Metabolic dysregulation and obesity

(Altered BA signaling affects glucose homeostasis, lipid metabolism, and energy balance).

Metabolic syndrome encompasses a cluster of interrelated metabolic abnormalities, including obesity-associated insulin resistance, impaired glucose homeostasis, dysregulated lipid metabolism, and chronic inflammation driven by pro-inflammatory immune mediators.^78^ Its pathogenesis is multifactorial, involving genetic predisposition, dietary patterns, and environmental influences, with visceral obesity representing the most severe phenotype and conferring the greatest risk for the onset and progression of metabolic diseases. Among these risk factors, obesity, particularly visceral or central obesity, plays a central role, closely related to the initiation and progression of various metabolic diseases.^79^ Recent epidemiological data indicate a rising global prevalence of metabolic syndrome and obesity, with approximately 20%-30% of adults worldwide and 33%-36% of adults in the United States affected.^80^^,^^81^

FXR is essential for glucose homeostasis, as whole-body FXR knockout mice exhibit elevated circulating free fatty acids (FFAs), impaired glucose and insulin tolerance hyperglycemia, and ultimately develop severe hepatic steatosis.^82^ It is also important to note that hepatic and intestinal FXR perform distinct yet interdependent functions in maintaining metabolic homeostasis. Hepatic FXR primarily governs BA synthesis and lipid metabolism by inducing SHP to repress CYP7A1 and CYP8B1, thereby preventing excessive BA accumulation and promoting hepatoprotection.^67^ It also suppresses hepatic lipogenesis through inhibition of Sterol Regulatory Element-Binding Protein 1c (SREBP-1c), contributing to improved hepatic insulin sensitivity and lipid clearance.^83^ Recent pharmacological evidence has demonstrated that systemic FXR agonists such as OCA and GW4064 primarily target both hepatic and intestinal FXR, exerting beneficial effects on glucose and lipid metabolism in both preclinical models and clinical trials.^84^^,^^85^ GW4064 significantly lowers blood glucose and enhances the insulin sensitivity in diabetic db/db and obese ob/ob mice.^86^^,^^87^ These above effects are mediated by FXR through the suppression of hepatic gluconeogenic gene expression like Phosphoenolpyruvate carboxykinase (PEPCK) and Glucose-6-phosphatase (G6Pase), as well as by promoting hepatic glycogen synthesis.^86^

In contrast, intestinal FXR acts as a nutrient- and microbiota-responsive sensor that coordinates postprandial signaling through induction of FGF15/19, which communicates with hepatic FGFR4/β-Klotho to repress gluconeogenesis and CYP7A1 expression.^68^ FGF15/19 inhibits hepatic gluconeogenesis by promoting the dephosphorylation and inactivation of cAMP response element-binding protein (CREB), thereby suppressing the expression of peroxisome proliferator-activated receptor *γ* coactivator-1α (PGC-1α) and other genes involved in hepatic glucose metabolism.^88^ Recent studies have identified intestine-restricted FXR agonists, such as fexaramine, that selectively activate intestinal FXR without significant hepatic exposure. Treatment with fexaramine induces intestinal FGF15 expression, enhances GLP-1 secretion, and promotes adipose tissue browning, collectively improving glucose tolerance, insulin sensitivity, and energy expenditure in obese mice.^89^^,^^90^ These beneficial effects are dependent on the gut microbiota, as antibiotic treatment abolishes them, accompanied by reduced levels of LCA-producing bacteria.^90^ Moreover, the gut microbiota shaped by gastric bypass surgery has been shown to reduce adiposity in diet-induced obese mice by enhancing energy expenditure in brown adipose tissue. This metabolic improvement is attributed to altered taurine metabolism and increased levels of taurine-conjugated BAs in the intestine and circulation, which subsequently activate intestinal FXR and systemic TGR5 signaling pathways, thereby promoting adaptive thermogenesis.^91^

However, the role of intestinal FXR in metabolic disorders remains controversial. Some studies have shown that gut microbiota altered by a Western diet can promote weight gain and hepatic steatosis in wild-type mice, but not in whole-body FXR knockout mice.^92^ These findings suggest that microbiota-induced obesity and steatosis are dependent on functional FXR, as whole-body FXR deficiency confers resistance against diet-induced metabolic disorders. Furthermore, they found that germ-free mice exhibit an enrichment of primary BAs, including elevated levels of tauro-*β*-muricholic acid (TβMCA), which acts as an antagonist of intestinal FXR and consequently diminishes the FXR-mediated suppression of hepatic BAs synthesis.^42^ Besides, short-term metformin treatment decreases *Bacteroides fragilis* and increases the FXR antagonist glycoursodeoxycholic acid (GUDCA) in the gut, thereby inhibiting intestinal FXR signaling and improving glucose metabolism, demonstrating that metformin’s metabolic benefits are mediated, in part, through the *B. fragilis*-GUDCA-intestinal FXR axis.^93^ More recently, a newly identified class of BA–methylcysteamine (BA-MCYs) conjugates, act as FXR antagonists and promotes BA biosynthesis. These host-derived conjugates were shown to reduce hepatic lipid accumulation in mice fed a high-cholesterol diet and counterbalance the FXR agonist effects of microbiota-derived free BAs, thereby fine-tuning BA signaling and maintaining hepatobiliary homeostasis.^33^ Collectively, these findings suggest that short-term inhibition of intestinal FXR may reduce fat absorption and yield metabolic benefits, such as decreased weight gain and improved glucose metabolism, primarily by modulating BA signaling and composition. However, the long-term consequences of sustained intestinal FXR inhibition remain uncertain, as chronic or genetic FXR deficiency has been associated with increased intestinal tumorigenesis.^94^^,^^95^ Additionally, prolonged FXR activation can disrupt cholesterol metabolism, potentially leading to increased plasma low-density lipoprotein (LDL) cholesterol and decreased high-density lipoprotein (HDL) cholesterol.^96^ Besides the divergent roles between hepatic and intestinal FXR, different routes of administration have opposite effects. One typical example is GW4064, which can activate both hepatic and intestinal FXR.^97^ Administration of GW4064 via the intraperitoneal route showed metabolic beneficial effects on mice fed with either HFD or high-fat and high-cholesterol diet.^98^ However, oral administration of GW4064 led to a pronounced worsening of the changes in liver and adipose tissue.^85^ These contrasting outcomes reflect pharmacokinetic differences: oral gavage involves gastrointestinal absorption and first-pass hepatic metabolism, producing slower onset and prominent gut–liver interaction, while intraperitoneal injection bypasses the gut, yielding faster systemic absorption, higher bioavailability, and limited intestinal exposure.^9^

Therefore, the therapeutic application of FXR agonists or antagonists in metabolic dysregulation requires careful optimization of both tissue selectivity and administration routes to balance metabolic benefits against potential lipid-related or tumorigenic risks.

In addition to FXR, the gut microbiota plays a key role in regulating GLP-1 secretion through TGR5. When the microbiota is depleted, the normal post-meal GLP-1 response disappears, but it can be restored by fecal microbiota transplantation or by adding specific BAs, such as *ω*-muricholic acid (ωMCA), hyocholic acid (HCA), or hyodeoxycholic acid (HDCA), which activate TGR5 in intestinal L cells.^100^^,^^101^ HCA also inhibits intestinal FXR, lifting its suppression of proglucagon transcription and further boosting GLP-1 production.^101^ Table 1 summarized the BAs on metabolic dysregulation and obesity.

**Table 1.** Summary of the BAs on metabolic dysregulation and obesity.

BA	Receptor	Disease	Downstream pathway	Outcome	Ref
GUDCA	FXR (Antagonist)	Obesity	Antagonize intestinal FXR to induce thermogenic and insulin-sensitizing genes in white adipose tissue	Ameliorate insulin resistance	[93]
BA-MCY conjugates, like CDCA-MCY	FXR (Antagonist)	Hypercholesteremia	Antagonize intestinal FXR and promote expression of BA biosynthesis genes in vivo	CDCA-MCY greatly decreases hepatic lipid accumulation in high-cholesterol diet (HCD)-fed mice.	[33]
HCA	FXR (Antagonist) TGR5 (Agonist)	Diabetes	Antagonize intestinal FXR and activate TGR5 to promote GLP-1 secretion	Maintains glucose homeostasis	[101]
	TGR5 (Agonist)	unknown	Modulate postprandial GLP-1 dynamics through enteroendocrine signaling	Maintain the physiological GLP-1 level	[102]
ωMCA	TGR5 (Agonist)	unknown	Modulate postprandial GLP-1 dynamics through enteroendocrine signaling	Maintain the physiological GLP-1 level	[102]

BAs are listed in the same order as in the manuscript.

### 3.2. Fibrosis and chronic liver diseases

Liver diseases, including fatty liver disease, biliary cholangitis, and liver cancer, account for approximately 4% of all deaths globally.^102^ Excessive accumulation of lipids and BAs in the liver disrupts metabolic and cellular homeostasis, fostering a pro-inflammatory and fibrogenic milieu that promotes the progression of MAFLD, MASH, fibrosis, cirrhosis, and HCC. FXR serves as a central regulator of BAs homeostasis, lipid metabolism, and inflammatory responses in the liver. Dysregulation of FXR signaling is closely associated with the onset and advancement of liver diseases, making FXR an attractive therapeutic target for restoring BAs balance, reducing hepatic steatosis, and mitigating liver inflammation and fibrosis.^103^^,^^104^

Increasing recognition of the gut-liver axis has underscored the pivotal role of gut microbiome-derived signals, particularly BAs, in liver pathophysiology. Dysregulated BA homeostasis, characterized by excessive hepatic BA accumulation, altered BA pool composition, and impaired microbial BA transformation, impairs lipid metabolism and insulin sensitivity, fostering metabolic dysfunction and hepatic steatosis.^45^ For example, patients with MAFLD and MASH display elevated levels of DCA and reduced levels of CDCA, along with shifts in the gut microbiome composition.^105^ These changes result in diminished hepatic FXR activation and impaired FGFR4-mediated feedback inhibition of BAs synthesis.^105^ Elevated secondary BAs such as DCA and LCA contribute to disease progression by promoting hepatocyte injury, oxidative stress, inflammation, and fibrosis. In contrast, other BAs like UDCA, TUDCA or GUDCA forms are anti-inflammatory and cytoprotective, improving glucose metabolism and reducing hepatic steatosis.^106^ HDCA have been shown to enhance GLP-1 secretion, promote TGR5 signaling, and improve metabolic and hepatic outcomes in experimental models of MAFLD, by inhibiting FXR, upregulating hepatic CYP7B1 and PPARα signaling.^107^ In addition, a recent study identified 3-succinylated cholic acid (3-sucCA), a microbial-derived BA produced by *Bacteroides uniformis*, which alleviates MASH by promoting the growth of *Akkermansia muciniphila* and is negatively correlated with liver damage in MAFLD patients.^29^ Of note, the therapeutic efficacy of OCA in MAFLD and NASH is influenced by the gut microbiome, which modulates BAs metabolism.^108^ Specifically, OCA administration enriches microbial populations encoding 7α-HSDHs enzymes responsible for converting primary to secondary BAs, rather than microbes enriched in BSH, which mediate BAs deconjugation.^108^^,^^109^

Epigenetic alterations are implicated in MAFLD pathogenesis, where aberrant DNA methylation affects genes controlling BA and xenobiotic metabolism, including CYP27A1, OSTα, SLC27A5, SLCO2B1, SLC47A1, and multiple CYP and UGT family members.^110^ Beyond receptor signaling, BAs also regulate hepatic epigenetic programs that maintain BA and metabolic homeostasis. Lysine-specific histone demethylase 1 (LSD1), which is directly induced by FXR and recruited in an SHP-dependent manner to the promoters of CYP7A1, CYP8B1, and NTCP, removes the activating histone marker H3K4me3. This initiates repressive chromatin remodeling characterized by H3K9/K14 deacetylation and H3K9 methylation, thereby silencing BA synthesis and uptake genes. Hepatic LSD1 deficiency results in elevated BA concentrations and hepatic inflammation, highlighting LSD1 as a crucial FXR/SHP-dependent epigenetic repressor that protects the liver from BA toxicity.^111^ Moreover, TUDCA, a weak FXR modulator, remodels histone methylation patterns, dimethylated H3K4, H3K27, and H3K36, at the promoters of Cell Death-Inducing DNA Fragmentation Factor-Like Effector A (Cidea) and C (Cidec) genes in offspring exposed to maternal undernutrition, down-regulating their expression and alleviating hepatic lipid accumulation even under high-fat diet conditions.^112^

Beyond MAFLD, cholestatic liver diseases such as primary sclerosing cholangitis (PSC) and primary biliary cholangitis (PBC) are increasingly recognized as disorders of gut microbial and BA dysregulation.^113^ Patients with PBC and PSC exhibit reduced microbial diversity, decreased abundance of beneficial taxa such as *Faecalibacterium* and *Lachnospiraceae*, and increased levels of potentially pathogenic bacteria, including *Enterobacteriaceae* and *Veillonella*.^114^^,^^115^ These microbial changes are associated with disturbed BA profiles, notably elevated levels of conjugated primary BAs and a reduction in secondary BAs, reflecting impaired microbial BA transformation. In murine models of PBC, disruption of the gut microbiota impairs the negative feedback regulation of BAs synthesis, resulting in elevated hepatic BA concentrations, compromised bile duct barrier integrity, and exacerbated liver injury, effects attributed in part to impaired FXR signaling and dysregulation of CYP7A1.^116^ Furthermore, *Prevotella copri* has been found to be significantly reduced in the gut microbiota of PSC patients.^117^ Restoration of *Prevotella copri* in PSC mouse models significantly improved cholestasis and liver fibrosis, likely through enhancement of FXR-mediated signaling pathways.^118^

Similarly, chronic hepatitis B patients with moderate to advanced fibrosis exhibit elevated serum levels of total and primary BAs compared to healthy controls, while fecal total and secondary BA levels are significantly reduced.^119^^,^^120^ This shift in BAs distribution is accompanied by enhanced activation of intestinal FXR by microbially BAs, resulting in increased serum levels of FGF19, which subsequently suppresses the primary BAs synthesis through downregulating hepatic CYP7A1 expression.^119^

Together, these findings highlight the multifaceted roles of the BA-FXR-microbiota axis in hepatic fibrosis and chronic liver diseases. While animal studies support the metabolic benefits of intestine-specific FXR knockout or FXR antagonism, the long-term consequences, particularly regarding BA homeostasis and liver-gut health, remain unclear. Further research is needed to delineate the precise contribution of intestinal FXR inhibition to these benefits and to determine whether such approaches can be translated into safe, long-term therapies for MAFLD and MASH in humans. Conversely, although FXR agonists (e.g. OCA) have demonstrated clinical potential for these indications, systemic activation is associated with adverse effects such as pruritus, dyslipidaemia, and possible negative impacts on cholesterol and cardiovascular risk.^96^^,^^121^ Together, these concerns underscore the importance of context- and tissue-specific modulation of the gut-liver axis in the development of future therapeutic strategies for these liver diseases. We summarized the mentioned BAs in Table 2.

**Table 2.** Summary of the BAs on liver diseases.

BA	Receptor	Disease	Downstream pathway	Outcome	Ref
DCA	FXR (Antagonist)	MAFLD	Antagonize hepatic FXR and suppress FGFR4 signaling, altering bile acid-lipid crosstalk	Unknown	[106]
CDCA	FXR (Agonist)	MAFLD	Activate hepatic FXR to modulate bile acid synthesis and lipid metabolism		
HDCA	FXR (Antagonist)	MAFLD	Antagonize hepatic FXR and activate CYP7B1 and PPARα to enhance lipid catabolism	Ameliorate hepatic steatosis	[108]
3-sucCA	Unknown	MASH	Promote the growth of *Akkermansia muciniphila*	Alleviate MAFLD-MASH progression	[29]
TUDCA	FXR (Agonist)	Hepatic steatosis	Activate FXR and remodels epigenetic dimethylation of H3K4, H3K27, and H3K36 to regulate metabolic gene expression	Alleviate hepatic fat deposition	[113]

BAs are listed in the same order as in the manuscript.

### 3.3. Inflammation and immune regulation

The FXR-BAs axis plays a critical role not only in metabolic regulation but also in modulating intestinal inflammation, infection susceptibility, and systemic immune responses. IBD, encompassing Crohn’s disease and ulcerative colitis, is characterized by chronic intestinal inflammation and significantly elevates the risk of CRC in patients with long-standing colitis.^122^ Although the precise etiology of IBD remains unclear, mounting evidence implicates gut microbiota dysbiosis as a key pathogenic factor.^123^ This microbiome imbalance significantly impacts BAs metabolism, which is increasingly recognized as a critical driver of mucosal inflammation.^3^^,^^124^ IBD-associated dysbiosis is characterized by increased levels of primary BAs (e.g., CA, TCA, GCA, CDCA, and GCDCA), and a marked reduction of secondary BAs (e.g., LCA and DCA), reflecting the loss of bacterial taxa responsible for BAs biotransformation.^76^^,^^125^

In genetically predisposed individuals, IBD is driven by an abnormal activation of both innate and adaptive immune responses targeting the intestinal microbiota. Early in IBD, compromised epithelial barrier integrity allows microbes’ translocation across the mucosa,^126^ triggering innate immune activation. Dendritic cells (DCs), macrophages, IECs, and (to a lesser extent) myofibroblasts recognize conserved microbial structures known as pathogen-associated molecular patterns (PAMPs) through pattern recognition receptors (PRRs), including Toll-like receptors (TLRs) and nucleotide-binding oligomerization domain 2 (NOD2), thereby initiating pro-inflammatory signaling and the release of cytokines such as interleukin-1 beta (IL-1β), tumor necrosis factor *α* (TNFα), and IL-6.^127^ Subsequently, the adaptive immune system is engaged, marked by heightened T helper 1 (Th1) and Th17 responses, or in some cases, a skewed Th2 profile.^128^ Dysregulated T cell differentiation and activation lead to excessive cytokines and chemokines production, fueling chronic inflammation and tissue injury.^128^ Lymphocytes, including CD4⁺ helper and CD8⁺ cytotoxic T cells, coordinate protective mucosal immune responses, while regulatory T cells (Tregs) maintain tolerance and suppress excessive inflammation.^129^ Forkhead box protein P3 (FOXP3)^+^ Treg cells are essential for intestinal immune homeostasis, with approximately 65% of colonic and 35% small intestinal Tregs expressing RORγt.^130^ RORγt drives Th17 cell differentiation and proinflammatory IL-17 production, while Treg-derived IL-10 counteracts Th17-mediated inflammation to preserve mucosal integrity.^131^^,^^132^

Microbial-derived BAs, including LCA, DCA, and their derivatives such as iso-, 3-oxo-LCA/DCA, allo-, 3-oxoallo-, and isoalloLCA, recently have been reported as key signaling molecules that modulate intestinal immune responses.^28^^,^^62^^,^^133^ For example, 3-oxo-LCA and 3-oxo-DCA act as RORγt antagonists, inhibiting its function and thereby suppressing Th17 cell differentiation, while isoalloLCA promotes Treg cell differentiation by inducing mitochondrial reactive oxygen species production, which enhances FOXP3 expression.^62^^,^^134^ Additionally, isoalloLCA augments Treg development by facilitating NR4A1 binding to the Foxp3 locus, thereby increasing Foxp3 transcription.^28^ Moreover, DCA negatively regulates CD8^+^ T cell effector function, by targeting plasma membrane Ca^2+^ ATPase (PMCA) to inhibit Ca^2+^-nuclear factor of activated T cells (NFAT) signaling.^133^ Notably, another study showed that *β*-hydroxydeoxycholic acid (3-epi-DCA, CAS Number: 570-63-8) promotes Foxp3 expression by reducing the immunostimulatory capacity of DCs.^135^ Importantly, FXR deletion in DCs enhances Treg differentiation and produces a gene expression profile similar to that induced by 3-epi-DCA, indicating a functional link between 3-epi-DCA and FXR in DCs.^135^

Beyond the direct effects of microbial BAs on immune cells, BA receptors are crucial regulators of intestinal inflammation, acting indirectly by preserving mucosal integrity through modulation of the BAs pool, or by directly modulating immune cell development and activation. FXR, in particular, has been shown to protect against IBD. Loss of FXR leads to increased intestinal expression of pro-inflammatory cytokine genes,^136^ while pharmacological activation of FXR with OCA prevents dextran sodium sulfate (DSS) and 2,4,6-trinitrobenzene sulfonic acid (TNBS)-induced intestinal inflammation, likely by protecting mucosal integrity, thus suppressing pro-inflammatory cytokines such as TNFα.^137^ Likewise, the administration of CDCA and LCA in TNB-induced colitis mice improved intestinal barrier integrity and reduced inflammation, primarily through the activation of the FXR and TGR5.^138^ Interestingly, another study found that treatment with TUDCA, an antagonist or a weak agonist of FXR, ameliorated ileitis in mice via enhancing FXR signaling.^139^

Notably, one study showed that FXR is intrinsically expressed in ILCs and serves as a key regulator of ILC3 development and function during intestinal inflammation. Activation of FXR in ILC3s by the gut-selective agonist fexaramine suppresses pro-inflammatory cytokine production and protects against colitis in multiple IBD mouse models, whereas FXR deletion in ILC3s exacerbates intestinal inflammation.^140^ These results establish FXR as a pivotal intrinsic modulator of ILC3-mediated immune responses, highlighting the link between BAs signaling, innate immunity, and intestinal homeostasis. Additionally, a recent study showed that loss of FXR in gut macrophages heightens pro-inflammatory cytokine production and exacerbates intestinal inflammation, while activation or gain of FXR function in these cells suppresses inflammation by modulating macrophage recruitment, polarization, and immune interactions.^141^ These results identify macrophage-intrinsic FXR as a promising therapeutic target for IBD. In addition to FXR’s role in NLRP3-related inflammatory diseases, the activation of TGR5 by TLCA stimulates PKA, which promotes the phosphorylation and ubiquitination of NLRP3, thereby inhibiting NLRP3 inflammasome-mediated inflammation in macrophages and mice.^142^

Beyond direct effects of microbial BAs, the gut microbiota also influence immune cell populations via reshaping BAs. Germ-free mice have reduced colonic RORγ⁺ Treg cells, but colonization with *Bacteroides thetaiotaomicron* and *Bacteroides fragilis* restores these cells by activating VDR and FXR signaling.^50^ However, disruption of BSH in these bacteria significantly impairs this effect.^50^ Furthermore, oral administration of a defined bacterial consortium – including *Clostridium AP sp000509125*, *Bacteroides ovatus*, and *Eubacterium limosum* – restored secondary BAs metabolism in DSS-treated mice, increasing UDCA and LCA levels.^143^ This consortium, in turn, activated TGR5, improved intestinal barrier integrity, and reduced inflammation in colitis models.^143^ Moreover, administration of *Bacteroides uniformis* to DSS-treated mice altered nuclear factor kappa B (NF-κB) and mitogen-activated protein kinase (MAPK) signaling and affected Th17 cell differentiation in the colon. However, *Bacteroides uniformis* did not directly inhibit Th17 cell differentiation *in vitro.*^144^ Instead, it influenced the intestinal immune response *in vivo* by modulating BAs metabolism and changing the levels of key metabolites such as *α*-MCA, HDCA, and isoLCA.^144^ Furthermore, mice colonized with microbiota from severe ulcerative colitis patients showed increased intestinal inflammation, fewer ILC3s, and reduced colonic VDR and PXR expression. Notably, 12-keto-LCA had strong anti-inflammatory effects by boosting VDR expression and lowering IL-17A secretion from ILC3s.^145^ A summarized table of BAs on inflammation and immune regulation is shown in Table 3.

**Table 3.** Summary of the BAs on inflammation and immune regulation.

BA	Receptor	Disease	Downstream pathway	Outcome	Ref
3-oxo-LCA	RORγt (Antagonist)	IBD	Antagonize RORγt signaling to inhibit Th17 cell differentiation	Suppress intestinal inflammation	[62]
3-oxo-DCA	TGR5 (Agonist)	Colitis	Activate TGR5 and inversely modulate RORγt to rebalance macrophage and Th17/Treg responses	Reverse dysbiosis and intestinal inflammation	[135]
	RORγt (Inverse agonist)				
isoalloLCA	RORγt (Antagonist)	IBD	Induce mitochondrial reactive oxygen species (ROS) to increase FOXP3 expression and Treg differentiation	Promote Treg generation	[62]
	NR4A1 (Antagonist)	IBD	Facilitate a permissive chromatin structure at the Foxp3 promoter	Enhance Treg cells differentiation	[28]
DCA	unknown	CRC	Diminish Ca²⁺-NFAT2 signaling by potentiating PMCA activity	Prevent CRC development	[134]
3-epi-DCA	FXR (Antagonist)	unknown	Antagonize FXR and enhances Foxp3 induction in dendritic cells	Enhance Treg cells generation	[136]
CDCA	FXR and TGR5 (Agonist)	Colitis	Activate FXR and TGR5 to strengthen gut barrier integrity and suppress pro-inflammatory cytokines	Improve gut barrier function and attenuate colitis	[139]
LCA					
TUDCA	unknown	Crohn’s disease	Stabilize bile acid homeostasis and mitigate endoplasmic reticulum stress under inflammation	Protect BA homeostasis under inflammation	[140]
TLCA	TGR5 (Agonist)	Inflammation	Activate TGR5 to increase NLRP3 phosphorylation and ubiquitination	Mitigate LPS-induced systemic inflammation and alum-induced peritoneal inflammation	[143]
12-keto-LCA	VDR (Agonist)	Ulcerative colitis	Activate VDR to suppress IL-17A expression from colonic ILC3	Ameliorate DSS-induced acute colitis	[146]

BAs are listed in the same order as in the manuscript.

### 3.4. Infectious diseases

Infectious diseases are disorders caused by pathogenic organisms such as bacteria, viruses, fungi, or parasites. Emerging evidence suggests that the BA-FXR axis plays a crucial role in host defense and immune regulation during infection.

*Clostridioides difficile* (formerly *Clostridium difficile*), commonly known as *C. difficile*, is a Gram-positive, anaerobic, spore-forming bacterium responsible for CDI.^146^ Its spores exhibit remarkable resistance to heat and numerous disinfectants, contributing to the persistence and transmission of CDI. Infection typically manifests as diarrhea and may progress to severe, life-threatening colitis.^147^ CDI is typically treated with antibiotics, however, such therapy disrupts the gut microbiota, thus increasing the susceptibility to recurrent CDI (rCDI).^148^The BAs-FXR axis also plays a role in CDI. BAs have been reported to show different impacts on *C. difficile*. TCA promotes the germination of *C. difficile,*^149^ while CDCA inhibits the germination and growth.^150^ In addition, antibiotic-induced gut dysbiosis disrupts the BAs pool and prevents the conversion of primary to secondary bile acids, thereby facilitating spore germination.^151^ Fecal microbiota transplantation (FMT) has emerged as the most effective therapy for rCDI, with cure rates exceeding 90%.^152^ FMT restores the functional microbial network capable of bile acid biotransformation, re-establishing the balance between primary and secondary bile acids. Post-FMT analyses demonstrate markedly elevated levels of secondary bile acids (such as DCA and LCA) and reduced levels of conjugated primary bile acids, accompanied by up-regulation of intestinal FXR signaling and increased circulating FGF19, a downstream FXR target.^153^^,^^154^ Besides the direct inhibitory effect on *C. difficile* germination and vegetative growth,^155^ UDCA pretreatment can reduce early CDI severity by activating FXR and TGR5 and modulating NF-κB-mediated immune responses.^156^

The role of BA signaling is also evident in *Staphylococcus* *aureus* infection. This pathogen can cause life-threatening diseases, including mastitis. Infected cows show reduced levels of CA and DCA compared to healthy controls.^157^ Notably, DCA, but not CA, was effective in alleviating *Staphylococcus aureus*-induced mastitis in mice by enhancing blood-milk barrier integrity and suppressing inflammation, which was mediated by DCA-induced TGR5 activation to inhibit NF-κB and the NLRP3 inflammasome signaling pathway.^157^ Moreover, the BA-FXR axis contributes to host responses during SARS-CoV-2 infection. Elevated temperature increases DCA production, which activates FXR and TGR5 to suppress virus replication and neutrophil-related tissue damage, protecting hamsters against severe COVID-19.^158^ However, FXR inhibition by UDCA downregulates ACE2 in human lung tissue, potentially lowering viral entry and infection risk.^159^ Table 4 shows a summary of BAs on infectious diseases.

**Table 4.** Summary of the BAs on infectious diseases.

BA	Receptor	Disease	Downstream pathway	Outcome	Ref
UDCA	FXR and TGR5 (Agonist)	Clostridioides difficile infection (CDI)	Activate FXR/TGR5 signaling to modulate NF-κB signaling and cytokine transcription	Attenuate intestinal inflammation and improve mucosal recovery	[157]
DCA	TGR5 (Agonist)	*Staphylococcus aureus* infection	Activate cAMP-PKA signaling to inhibit NF-κB and NLRP3 inflammasome signaling	Alleviate *Staphylococcus aureus* -induced mastitis	[158]
DCA	TGR5 (Agonist)	Influenza A virus	Activate TGR5 to suppress viral replication and limit neutrophil-mediated tissue damage	Increase host resistance to influenza A infection	[159]
	FXR (Agonist)	SARS-CoV-2	Unknown	Improve host survival rate	
UDCA	FXR (Antagonist)	SARS-CoV-2	Antagonize FXR to downregulated ACE2 expression in human airway and lung tissue	Reduce SARS-CoV-2 entry and infection	[160]

BAs are listed in the same order as in the manuscript.

### 3.5. Tumorigenesis and cancer progression

Emerging evidence links the gut microbiota-BAs axis to the risk and progression of gastrointestinal cancers, including HCC and CRC. Dysregulated BAs homeostasis and impaired FXR signaling are closely associated with HCC initiation and progression. Clinical studies show that patients with HCC have markedly altered circulating BA profiles, typically with elevated primary conjugated BAs.^160^ Inhibition of conjugated BA synthesis in hepatocytes, achieved through deletion of the BA-conjugating enzyme BAAT enhances tumor-specific CD8⁺ T-cell responses, suppresses tumor growth, and sensitizes tumors to anti-programmed cell death-1 immunotherapy.^160^ LCA inhibits T cell function through endoplasmic reticulum stress to increase tumor nodules, which could be countered by UDCA through boosting CD8 ^+^ T cell cytotoxity.^160^ However, specific BA species such as DCA can accumulate in HCC depending on disease stage or tissue compartment, and several studies demonstrate that elevated DCA levels promote hepatocarcinogenesis through oxidative stress, DNA damage, and senescence-associated secretory phenotype (SASP) induction.^161^ Moreover, one study found that HCC-bearing mice have increased levels of TβMCA and TCA; however, TβMCA’s strong FXR antagonism overrides TCA’s agonism, leading to hepatic FXR inhibition.^162^ This drives activation of the Yes-associated protein (YAP) transcriptional enhanced associate domain (TEAD) pathway and promotes HCC, highlighting the crucial role of BAs-FXR balance in liver cancer development.^162^

Loss of FXR further disrupts BAs metabolism and increases HCC risk, as whole-body FXR knockout mice spontaneously develop spontaneous liver tumors due to elevated BAs levels, supporting FXR’s tumor-suppressive role.^163^ Either hepatocyte-specific or intestine-specific FXR deficiency alone does not lead to liver tumorigenesis, indicating that FXR loss acts as a tumor initiator, with dysregulated BAs hemostasis required for full tumor development.^164^ Importantly, pharmacological activation of FXR with agonists like OCA and GW4064 has been shown in animal studies to suppress tumor growth and inflammation while restoring BAs balance, thereby improving HCC outcomes.^165^^,^^166^ Furthermore, restoring FXR activity specifically in intestinal enterocytes corrects BAs balance and prevents spontaneous HCC in FXR-null mice by reducing BAs production in the liver.^167^

CRC is often associated with altered gut microbiota composition and function, leading to impaired deconjugation and transformation of BAs.^168^ This results in higher circulating and fecal levels of conjugated primary BAs, such as GCA, TCA, GCDCA, TCDCA, and GHCA, and frequently a reduction in deconjugated secondary BAs like GDCA and TDCA.^169^^,^^170^ while toxic secondary BAs like DCA may be increased in some cases.^171^ Additionally, BSH-expressing *Bacteroides* can increase unconjugated BAs in the colon and, under HFD conditions, promote CRC progression through TGR5 activation mediated by DCA and LCA.^172^

FXR has emerged as a potential tumor suppressor in CRC. 3-oxo-LCA, has been identified as an FXR agonist to induce apoptosis to inhibit CRC tumorigenesis and progression.^173^ Mice lacking FXR are more susceptible to intestinal tumorigenesis,^95^^,^^174^ and diminished FXR activity is observed in CRC patients.^175^^,^^176^ Mechanistically, FXR inhibits Wnt signaling through direct interaction with *β*-catenin, thereby suppressing oncogenic pathways.^94^ Furthermore, the loss of FXR activation, potentially due to the presence of antagonistic BAs such as T-βMCA or elevated DCA levels, facilitates the malignant transformation of intestinal stem cells and accelerates adenoma-to-carcinoma progression.^174^ Moreover, effective modulation of FXR activity in macrophages by BAs is required to suppress the colitis-associated CRC, as FXR activation alleviates intestinal inflammation and inhibits tumor development by regulating macrophage recruitment, polarization, and their interactions with Th17 cells.^141^

Furthermore, CRC-promoting factors such as an HFD can alter the balance of BAs that either activate or inhibit FXR. For instance, 7-oxo-deoxycholic acid (7-oxo-DCA) acts as a neutral FXR antagonist and promotes tumor development, whereas isodeoxycholic acid (isoDCA, CAS Number: 566-17-6) serves as a potent FXR agonist and suppresses intestinal tumorigenesis.^39^ Future studies should investigate how bacteria with 7α/β-HSDH activity, which produce 7-oxo-DCA and isoDCA, compete and interact in vivo to influence CRC development, and explore strategies to modulate these microbial populations to prevent or inhibit CRC.

Beyond receptor-mediated signaling, BAs can influence host epigenetic landscapes to modulate tumorigenesis. In colon cancer cells, UDCA, an FXR antagonist, directly modulates chromatin, driving histone hypoacetylation and inducing differentiation or senescence.^177^ UDCA also restrains malignant progression in vivo by engaging the TGR5-cAMP/PKA-RhoA-YAP axis, suppressing YAP activity in AOM/DSS CRC models.^178^ DCA has been linked to HDAC6-dependent epigenetic remodeling in the gastrointestinal epithelium, and HDAC6 is overexpressed and pro-tumorigenic in CRC, supporting a BA-HDAC6 inflammatory/oncogenic route relevant to the lower gut as well.^179^^,^^180^ While direct modulation of histone modifications remains an emerging research area, this perspective is now included in the discussion. A summarized table of BAs on tumorigenesis and cancer progression is shown in Table 5.

**Table 5.** Summary of the BAs on tumorigenesis and cancer progression.

BA	Receptor	Disease	Downstream pathway	Outcome	Ref
LCA	unknown	HCC	Induce endoplasmic reticulum stress to impairs T cell function	Increase tumor nodules	[161]
UDCA	unknown	HCC	Boost CD8^+^ T cell cytotoxicity	Abolishe liver tumors	
DCA and LCA	TGR5 (Agonist)	CRC	Agonize TGR5 to induce Ccl28 expression in a *β*-catenin-dependent manner	Promote CRC progression	[174]
7-oxo-DCA	FXR (Antagonist)	CRC	Antagonize FXR to enhance WNT signaling	Promote intestinal tumorigenesis	[39]
isoDCA	FXR (Agonist)	CRC	Agonize FXR to suppress WNT signaling	Inhibit intestinal tumorigenesis	
3-oxo-LCA	FXR (Agonist)	CRC	Agonize FXR to induce apoptosis	Inhibit CRC tumorigenesis and progression	[174]
UDCA	FXR (Antagonist)	CRC	Antagonize FXR to induce histone hypoacetylation (chromatin remodulating)	Induce differentiation and senescence in colon cancer cells	[178]
UDCA	TGR5 (Agonist)	CRC	Agonize TGR5 to activate cAMP/PKA pathway toinhibit RhoA activity and YAP signaling	Inhibit colonic tumor growth	[179]
DCA	unknown	Gastric intestinal metaplasia	Upregulate HDAC6 in gastric epithelial cells to inhibit FOXP3 transcription	Increase intestinal metaplasia	[181]

BAs are listed in the same order as in the manuscript.

## 4. Therapeutic potential

The metabolic functions of BAs offer promising avenues for therapeutic intervention by directly or indirectly targeting the gut microbiota-BAs-BA receptor signaling axis in a range of diseases, including but not limited to metabolic, gastrointestinal, and immune-related diseases. Direct therapeutic strategies typically involve the administration of natural or synthetic BAs receptor agonists or antagonists (e.g., FXR or TGR5 modulators) to restore metabolic and immune balance. In contrast, indirect approaches focus on altering the composition or activity of the gut microbiota to reshape the BAs pool and downstream signaling. These methods include dietary interventions, prebiotics/probiotics, FMT, and emerging synthetic biology tools such as engineered bacteria capable of expressing key microbial enzymes, such as BSH or 7α-dehydroxylase. By influencing the complex interactions among the gut microbiome, BAs metabolism, and host receptors, these interventions offer a multi-pronged strategy to restore homeostasis and treat a variety of chronic and infectious diseases.

### 4.1. Pharmacological modulation of BA receptors

As discussed previously, UDCA, also known as ursodiol, has been used for decades in the treatment of liver diseases and was first approved by the U.S. FDA in 1987 for the dissolution of gallstones, and later in 1996 for primary biliary cirrhosis (now termed primary biliary cholangitis, PBC).^181^^,^^182^ However, approximately 40% of patients do not respond adequately to UDCA, resulting in higher rates of disease progression and mortality, underscoring the need for alternative or adjunctive therapies.^183^ Given the benefits of FXR activation in cholestatic liver diseases, FXR agonists have garnered considerable interest for PBC.^184^ CDCA was identified in 1999 as the most potent natural ligand for FXR. Building on this discovery, Roberto Pellicciari and colleagues designed, synthesized, and patented a series of alkylated BA analogs. Among these, OCA emerged as a highly potent FXR agonist ^185^ and received accelerated approval in the U.S. in 2016 for PBC patients unresponsive to or intolerant of UDCA.^186^ Moreover, OCA shows the potential to treat NASH, MAFLD, and related diseases.^187^^,^^188^ Despite the promising effects of OCA, its long-term side effects cannot be overlooked. In patients with PBC, 12 months of OCA, alone or with ursodiol, improved alkaline phosphatase, bilirubin, and other biochemical markers, but also increased pruritus in a dose-dependent manner, with the mechanism remaining unclear.^121^ Besides, in MASH patients, OCA therapy increased small very-low-density lipoprotein (VLDL) particles and both large and small LDL particles, while reducing HDL particles at 12 weeks; these changes returned to baseline 24 weeks after discontinuation.^96^ Therefore, precise dose adjustment or concomitant use of lipid-lowering agents should be considered to optimize the therapeutic benefit of OCA while mitigating adverse effects.

Moreover, exploring novel and promising BAs could be an alternative option. With advances in analytical technologies, a growing number of novel BA species have been identified and characterized. As discussed above, compounds such as BA-MCYs,^33^ amino acid-conjugated BAs (AA-BAs),^34^ isoDCA,^39^3-oxo-LCA,^173^ and 3-sucCA ^29^ are emerging as promising candidates for the development of small-molecule drugs targeting CRC, IBD, HCC, NASH, and other related diseases. BA-MCYs have been reported to act as intestinal FXR antagonists, reducing hepatic lipid accumulation in mice fed a high-cholesterol diet.^33^ These findings support the concept that intestinal FXR antagonists can alleviate hepatic steatosis in mice.^107^ AA-BAs activate FXR in vitro, and oral administration in mice reduce the expression of BA synthesis genes in vivo; however, their overall biological effects remain unknown.^34^ Our recent study demonstrates that activation of FXR by isoDCA or 3-oxo-LCA significantly inhibits CRC tumor initiation and progression in both mouse models and human CRC organoids, as well as in patient-derived xenograft models.^39^^,^^173^ 3-sucCA alleviated MASH in mice by promoting the growth of *Akkermansia muciniphila*, but whether this effect involves FXR or other receptors remains unknown.^29^ Though these compounds are promising, continued investigations into their molecular mechanisms, receptor specificities, and long-term safety will be essential for translating these compounds into clinically viable therapeutics.

### 4.2. Dietary and lifestyle interventions

Dietary intervention has been recognized as a safe and effective alternative to pharmacological treatment for obesity-related metabolic syndrome, and cancers.^189^^,^^190^ Among these strategies, caloric restriction has shown well-documented benefits, including significantly upregulating the expression of BA -synthetic and conjugating enzymes, as well as IBABP, thereby expanding the overall BA pool.^191^^,^^192^ These changes are associated with improvements in insulin sensitivity, *β*-cell function, and a reduced fat-to-body weight ratio.^193^ In addition to metabolic benefits, caloric restriction also suppresses tumor growth, enhances immune surveillance, and increases the sensitivity of cancer cells to chemotherapy.^194^ Notably, LCA has been reported to mimic the anti-aging effects of caloric restriction, suggesting a potential mechanistic link between BA signaling and dietary restriction.^195^

In addition to caloric restriction, modifying dietary composition may also offer therapeutic benefits. The ketogenic diet (KD), a high-fat, adequate-protein, low-carbohydrate dietary regimen, has traditionally been used in conventional medicine to treat refractory epilepsy in children.^196^ In recent years, however, accumulating evidence has highlighted its broader therapeutic potential, particularly in managing obesity-related metabolic syndrome and various types of cancer. KD has been shown to promote weight loss, improve lipid profiles, and enhance insulin sensitivity,^197^ which has been confirmed to relate to the increased level of TDCA and TUDCA.^198^ Moreover, in preclinical studies, KD has reduced tumor burden in animal models of malignant glioma, colon cancer, gastric cancer, and prostate cancer.^199^ However, another study has pointed out that the KD might trigger tumor metastasis by modulating BTB domain and CNC homolog 1-mediated transcription.^200^ Therefore, a deeper understanding of the interplay between KD and disease progression is warranted, especially in light of different physiological and pathological contexts. Notably, the impact of KD on BA metabolism, and reciprocally, the influence of BAs and their receptors (such as FXR, TGR5, and VDR) on the outcomes of KD, should be carefully examined in a disease-specific manner. This nuanced crosstalk may hold the key to both maximizing therapeutic efficacy and minimizing potential risks.

Beyond the macronutrient composition, incorporating BA-active nutrients, such as fibers that alter BA deconjugation and phytochemicals that modulate BA synthesis and transport, can be another promising strategy. Dietary fiber has been linked to BA deconjugation, likely through interactions with gut microbial BSHs and glycosidases in the small intestine.^201^ Polyphenols and polysaccharides have been reported to target FXR and TGR5, regulating BA synthesis and metabolism in a gut microbiota-dependent manner.^202^^,^^203^

Despite the promising benefits of dietary and lifestyle interventions, several shortcomings remain. First, inter-individual variability in response to nutrients is substantial, influenced by genetics, gut microbiota composition, age, sex, and underlying disease status.^204^ Such heterogeneity makes it difficult to predict which patients will benefit most from a given intervention. Second, many clinical studies are limited in duration and scale, raising concerns about the long-term safety and sustainability of these interventions. For instance, caloric restriction may not be feasible or well tolerated over extended periods,^205^ while KDs have been linked to adverse lipid changes and, induce cardiovascular disorders.^206^ Third, the mechanistic links between BAs, their receptors, and dietary interventions remain incompletely defined.

### 4.3. Microbiota-targeted therapies

FMT, the transfer of fecal microorganisms from a healthy donor to a patient, was originally developed to restore the protective functions of an intact gut microbiota, particularly in the treatment of CDI.^207^ Its therapeutic success in CDI is thought to be due, in part, to the restoration of BSH-dependent secondary BA production, which inhibits *C. difficile* growth, along with the activation of the bile acid-FXR-FGF signaling pathway.^153^^,^^207^ More recently, the application of FMT has expanded to a broader range of conditions, including colonic dysbiosis, metabolic syndrome, and chronic liver diseases.^208^^,^^209^ However, the role of BAs in mediating the therapeutic benefits of FMT in these conditions remains incompletely understood. Despite its promise, several limitations hinder the widespread adoption of FMT. Safety concerns remain paramount, particularly the risks of pathogen transmission, bacteriophage or antimicrobial resistance gene mobilization, and variability in donor screening stringency. Moreover, issues of durability and variability in microbial engraftment pose challenges for long-term efficacy. These concerns highlight the need for rigorous risk assessment and standardized protocols. To address these limitations, safer and more controlled alternatives to traditional FMT are under active investigation. These include the use of defined microbial consortia designed to recapitulate key ecological functions, pasteurized or pathogen-filtered FMT preparations, and spore-based microbiota products that reduce infection risk while maintaining therapeutic efficacy. Such strategies aim to harness the clinical benefits of microbiota transfer while improving safety, consistency, and regulatory acceptability.

Thus, compared with FMT, a more controlled and potentially safer alternative may be the development of specific bacterial therapies, such as probiotics or engineered strains with well-defined BA-metabolizing or -producing capabilities. For instance, genetically engineered *Clostridium spp.* with 7α-dehydroxylation activity have been shown to eliminate *C. difficile* due to their ability to produce inhibitory secondary BAs.^154^ Similarly, probiotics can be carefully selected or engineered for functional properties such as BSH activity, 7α-dehydroxylation capacity, or the ability to synthesize beneficial secondary BAs. These targeted manipulations of the BA pool can modulate host signaling pathways through receptors such as FXR, TGR5, and VDR. The probiotic mixture VSL#3, developed for the management of irritable bowel syndrome and ulcerative colitis, contains BSH-expressing bacterial strains that enhance BA deconjugation and promote BA excretion.^210^ Treatment with VSL#3 has been shown to upregulate hepatic BA biosynthesis by suppressing the FXR-FGF15 signaling pathway.^211^ In addition, VSL#3 restores insulin signaling and protects against nonalcoholic steatohepatitis and atherosclerosis in a model of genetic dyslipidemia and intestinal inflammation via direct transactivation of peroxisome proliferator-activated receptor-*γ*, FXR and VDR.^212^ Beyond VSL#3, candidate probiotics such as *Akkermansia muciniphila* and *Lactobacillus reuteri* have also been reported to reshape BA composition, improve intestinal barrier function, and ameliorate metabolic inflammation.^213^

A rapidly advancing direction is the use of engineered probiotics or designer microbial consortia to deliver targeted BA transformations, for example, controlled 7α-dehydroxylation or amino acid conjugation. Synthetic biology approaches now allow the introduction of kill-switches, auxotrophies, and other genetic safeguards to improve biocontainment and safety. Such strains could be designed to selectively enrich for protective secondary BAs (e.g., DCA, LCA derivatives) or to synthesize hepatoprotective species such as UDCA and TUDCA, while minimizing accumulation of hepatotoxic intermediates. Furthermore, modular consortia such as VE303 or synthetic spore-based therapeutics (e.g., SER-109) illustrate how defined combinations of BA-active microbes can be standardized, quality-controlled, and scaled for clinical use.^214^^,^^215^ In parallel, CRISPR-based editing and programmable gene circuits are enabling next-generation probiotics that respond dynamically to host BA levels, delivering therapeutic molecules only under disease-relevant conditions.

### 4.4. Systems biology and multi-omics integration

Systems-level approaches integrating multi-omics technologies have revolutionized our understanding of the BA–gut microbiota–host axis. By combining metagenomics, metabolomics, transcriptomics, proteomics, and lipidomics, it is now possible to delineate how microbial BA-transforming pathways and host receptor networks cooperate to maintain metabolic and immune homeostasis.

Integrative omics analyses have revealed that specific microbial and metabolic signatures can serve as non-invasive diagnostic biomarkers. A combined lipidomic–metaproteomic profile enables early detection of gallbladder cancer arising from biliary anomalies.^216^ Similarly, interrogation of shotgun metagenomic and untargeted metabolomic datasets using random forest modeling and differential-abundance analysis identified distinct microbial and metabolic signatures that accurately discriminate cirrhosis, underscoring their potential as promising non-invasive diagnostic tools.^217^ Beyond diagnostics, multi-omics analyses have uncovered physiological regulators of BA metabolism. For instance, integration of metabolomic and genomic data identified carboxylesterase 1c (Ces1c) as a key determinant of circulating TUDCA levels, demonstrating how systems-biology approaches can pinpoint molecular drivers of BA-linked metabolic traits.^218^

Comprehensive multi-omics investigations in liver and metabolic disorders have delineated disease-specific BA-microbiota interaction patterns. These include reduced microbial BSH and BAI gene abundance with accumulation of conjugated primary BAs in NAFLD/NASH, and enrichment of secondary BAs such as DCA and LCA associated with tumor-promoting inflammation and suppressed FXR activity in HCC and CRC.^219-221^ The integration of metagenomic and host transcriptomic data further delineates microbial-host co-regulatory networks involving FXR, TGR5, and PXR signaling across hepatocytes, enterocytes, and macrophages, supporting the existence of cross-tissue BA signaling circuits that shape metabolic tone and disease susceptibility. Importantly, these frameworks enable patient stratification, for example, identifying microbial and metabolic clusters predictive of response to FXR agonists (e.g., OCA) or BA-modulating therapies in MASH. Coupling multi-omics data with machine learning yields predictive biomarkers for distinguishing progressive versus non-progressive phenotypes. Crucially, a precision approach must consider the tissue-specific regulation of FXR. Indiscriminate FXR activation across the enterohepatic axis can produce opposing metabolic and immunologic outcomes depending on tissue context and microbial composition. Therefore, context-dependent and cell-selective FXR modulation, guided by integrated omics and spatial profiling, is essential to balance metabolic efficacy with immune and lipid homeostasis. Future applications integrating spatial metabolomics and single-cell multi-omics will permit mapping of BA-receptor signaling within specific tissue niches, ultimately leading to precision medicine strategies that harness BA-microbiota-host interactions for individualized prevention and therapy.

## 5. Concluding remarks

Mounting evidence underscores BAs as critical metabolic regulators and signaling molecules, playing significant roles in health and disease. The gut microbiota-BAs-BA receptor axis has emerged as a promising therapeutic target, providing valuable insights into BA-related pathologies and therapeutic opportunities. Clinically approved treatments such as UDCA and OCA, which modulate this axis, have demonstrated efficacy in cholestatic liver diseases, NASH, and IBD. Given the expanding relevance of the BA–FXR axis in conditions such as cancer, metabolic syndrome, and inflammatory disorders, extending the application of FXR agonists or antagonists, including OCA, warrants further exploration. However, tissue-specific and context-dependent FXR modulation remains a key challenge that requires deeper mechanistic and translational studies. Besides FXR, the biological functions of BAs primarily depend on interactions with host receptors, TGR5, VDR, CAR, and PXR, as well as their respective co-activators and co-repressors. These interactions are influenced by the structural specificity, concentration, bioavailability, and receptor expression profiles of individual BA species. Moreover, distinct BAs generated by gut microbes may trigger divergent downstream signaling cascades, suggesting receptor-dependent selectivity in cellular responses.

Despite notable progress, advancing the development of novel, safe, and effective BA-based therapies will require deeper foundational and translational insights. Crucially, future research should: 1. Clearly delineate hepatic versus intestinal FXR functions through inducible, tissue-specific FXR activation and deactivation models. Understanding tissue-specific FXR signaling will better define therapeutic windows and minimize off-target effects. 2. Systematically identify and characterize novel microbial-derived BA species, establishing their tissue-specific distribution, receptor affinities, and signaling biases. Clarifying these relationships will support the precise therapeutic exploitation of microbial BAs. 3. Investigate the mechanistic basis of divergent downstream signaling elicited by different microbial BAs. Understanding how distinct BA species engage differential receptor-associated signaling complexes may unlock new pathways for targeted therapeutic interventions. 4. Address inter-individual variability in gut microbiome composition and function, which significantly affects BA metabolism, bioavailability, and receptor engagement. Personalized therapeutic strategies may thus be developed to account for these differences, optimizing treatment efficacy. 5. Establish rigorous long-term safety and efficacy profiles of novel BA-based therapies through comprehensive preclinical and clinical trials, ensuring safe and sustainable therapeutic outcomes. Such insights will be essential for guiding the next generation of precision therapeutics targeting BAs signaling pathways.
